# The Periosteal Autografts Transplantation for Cartilage Defects of the Hip in Older Children With Developmental Dysplasia as an Adjunctive Procedure

**DOI:** 10.1097/MD.0000000000003432

**Published:** 2016-04-29

**Authors:** Ming-Hua Du, Yu Ding, Xian Shi, Rui-Jiang Xu

**Affiliations:** From the Department of Pediatric Orthopedic Surgery (M-HD, R-JX) and Department of Acupuncture and Moxibustion, Chinese PLA General Hospital (YD, XS), Beijing, China.

## Abstract

Cartilage lesions are at a high prevalence in dysplastic hips and may relate to arthritic changes and hip joint dysfunction. To date, the effectiveness of repair of articular cartilage defects in the dysplastic hips has not yet been thoroughly evaluated. Here we retrospectively reviewed the effects of acetabuloplasty procedures with/without concomitant autologous tibial periosteal transplantation (ATPT) for articular cartilage defects of the hip in older children with developmental dysplasia of the hip (DDH).

Older DDH children with focal cartilage defects of the acetabular or femoral cartilage or both in the hip joint were treated by acetabuloplasty procedures with (Group I) or without (Group II) concomitant ATPT to evaluate the improvements in range of motion (ROM), pain relief of hip, walking tolerability (WL), radiologic evaluations, and outcomes in the long-term follow-up.

More satisfactory functional outcome is readily achieved among patients treated with combined acetabuloplasty and ATPT, evidenced by marked pain relief and improved ROM and WL. The latest favorable radiologic evaluation was 70.6% in Group I and 60.0% in Group II, respectively. More hips exhibited congruency between the femoral head and the shell, with less deformity of femoral head and acetabulum or narrowed joint space in Group I. Few major complications were recorded in Group I.

Application of periosteal autograft for repair of cartilage defects within the hip joint might be an effective adjunctive treatment for acetabuloplasty in preventing stiffness, reducing pain, and improving ROM and outcomes in hip rehabilitation in the long-term follow-up in older children with DDH.

## INTRODUCTION

The treatment for neglected developmental dysplasia of the hip (DDH) in older children has constantly been a matter of controversy for pediatric orthopedists all over the world.^1,2^ Due to missed diagnosis or late presentation of syndromes, for cases of DDH in children aged over 8, not until syndromes of osteoarthritic changes appear, such as pain or functional limitation of the hip, would they come to visit the clinic. This makes the challenges of treating older DDH children lie in more perplexing pathological conditions: insufficiency of the acetabulum, higher displacement of the femoral head, contracted soft tissues and lesions of acetabular or femoral cartilage or both to a certain extent as well.

In spite of complex hip conditions and difficulties in surgical procedures for older DDH children, various acetabuloplasty techniques, such as shelf acetabuloplasty, Dega osteotomy, or Chiari osteotomy, have been well-established for possible reduction. Pathological conditions of acetabulum and femoral head and soft tissue release (STR) have been respectably evaluated in these patients. However, quality of cartilage in the prognosis of DDH remains unclear.

Cartilage lesions have been showed to be at a high prevalence in dysplastic hips,^3–6^ and the progression of cartilage abnormalities is highly related to severe pain, progressive stiffness, and dramatic loss of range of movement and function in hip anomalies.^6–11^ Hip cartilage defects might be one of the most elusive sources of hip joint pain and dysfunction.^12^ If left untreated, then arthritic changes may occur,^13^ but repair of the hip cartilage defects may relieve pain, restore function, and achieve promising clinical and radiological outcomes.^14^

Rising novel techniques in treating articular cartilage defects might encourage an adjunctive solution for the damaged hip. Several surgical techniques have been attempted to restore articular surfaces, including osteochondral transplantation,^15^ autologous chondrocyte implantation,^16^ and periosteal autografts transplantation.^17^ These reparative procedures are widely applied in the knee, but only a few case reports have been reported in hip.^18,19^ Periosteal transplantation is a well-established technique for the repair of cartilage lesions with promising results both in animal and human.^20–22^ The capacity of periosteum used as a free autologous graft to repair chondral defects in knee,^21,23^ shoulder,^24^ and even elastic ear^25^ is well documented. To date, however, application of periosteal autograft for the repair of hip cartilage defects as an adjunctive procedure for various acetabuloplasty techniques has not yet been evaluated in older children with DDH.

The purpose of this retrospective study is to report the effects of periosteal autograft transplantation for hip cartilage defects with concomitant various acetabuloplasty procedures for older DDH children on the improvements in range of motion (ROM), pain relief of hip, walking tolerability (WL), radiologic evaluations and outcomes and compare these results with similar cases treated by acetabuloplasty procedures alone to determine a better treatment protocol for older DDH children.

## MATERIALS AND METHODS

Group I included older DDH children (between 8 and 16 years old) with severe acetabular dysplasia or luxation or subluxation, and focal cartilage defects of the acetabular or femoral cartilage or both treated by acetabuloplasty and autologous tibial periosteal transplantation (ATPT) between January 1994 and December 2004. Group II included patients with similar demographic, clinical, and radiographic features at the time of diagnosis during the same time period but treated by acetabuloplasty alone. Signed informed consent was provided by all patients’ parents and approval was given by the ethics committee of our hospital.

The index procedures in this study were acetabuloplasty (with or without concomitant femoral osteotomy and STR [tenotomy of adductor and iliopsoas muscle and downward displacement of tendinous portion of the straight head of the rectus femoris muscle]) and ATPT (with in Group I, without in Group II) on hip management. The decision to perform certain acetabuloplasty was based on clinical and radiological signs, our senior surgeon's preference and parents’ selection when suggested.

Data on demographic details of gender, the side of involvement, previous treatment, age at the time of surgery, surgical procedure performed, location and size of hip cartilage defects, duration of follow-up, complications, need for subsequent operations and clinical and radiographic information preoperatively, postoperatively and at the latest follow-up were obtained from the hospital records and review of the radiography findings. The demographic data and clinical characteristics are shown in Table 1.

**TABLE 1:**
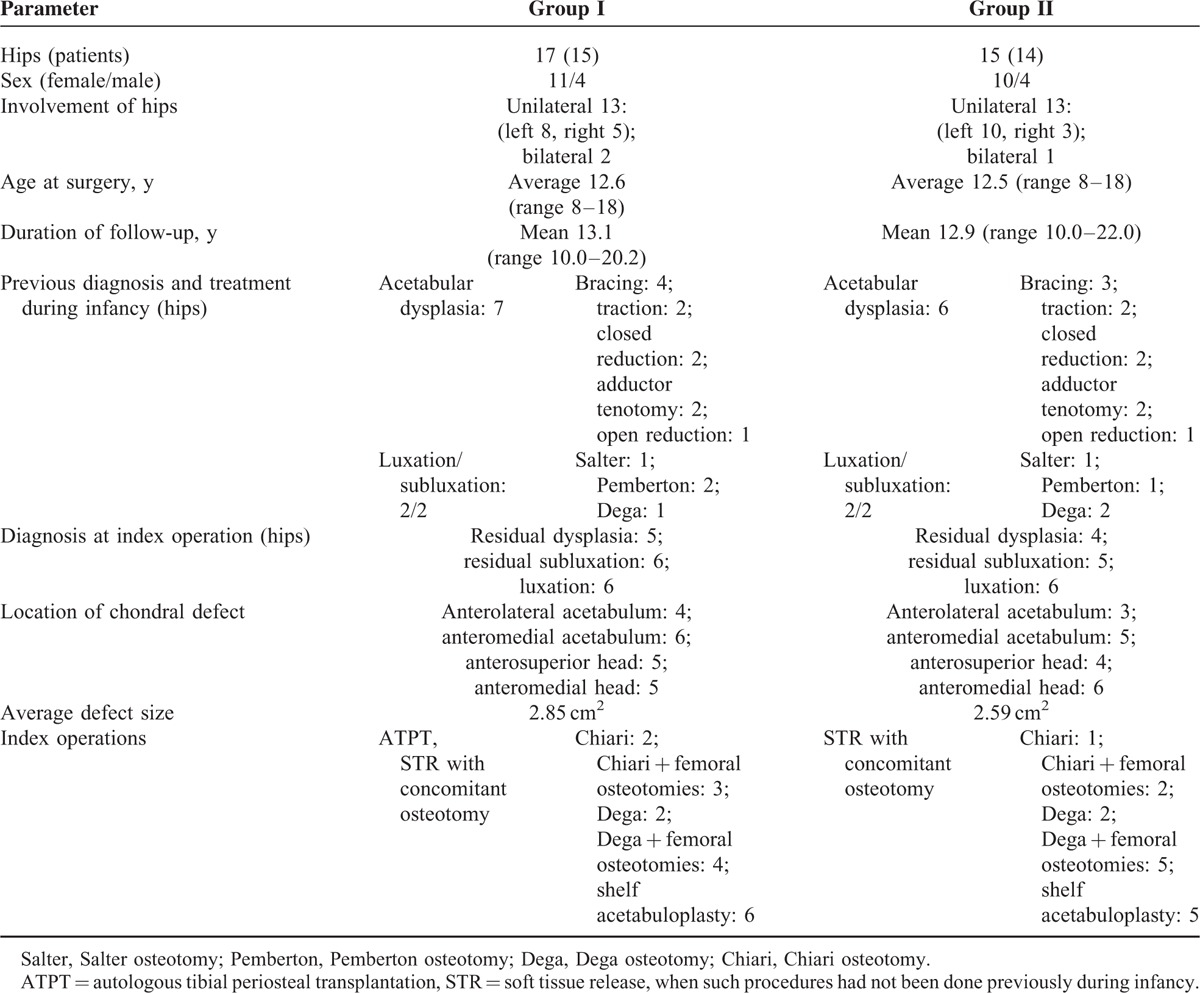
The Demographic and Clinical Information

Each patient's clinical status was assessed using an established scoring system preoperatively, at regular intervals after surgery, and the latest follow-up including criteria for pain, hip ROM, and level of ambulation (AL)^26^ (Table 2). A score of 0 on all 3 criteria indicated an excellent clinical result; a score of 1 or 2 on any of the 3 criteria indicated a good or poor result, respectively. Radiologic evaluations of the preoperative, postoperative, and final follow-up radiographs, were assessed for the joint space, center-edge (CE) angle, Sharp angle, Shenton line, and the modified Severin criteria^27^ (Table 3). And occurrence of complications was recorded postoperatively at follow-up on one's visiting at Dr. Xu Rui-Jiang's outpatient office.

**TABLE 2:**
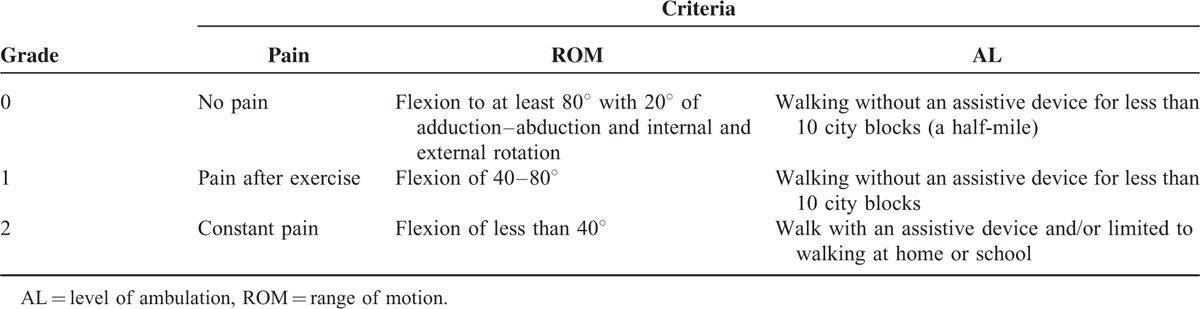
The Criteria for Evaluation of Pain, ROM, and AL

**TABLE 3:**
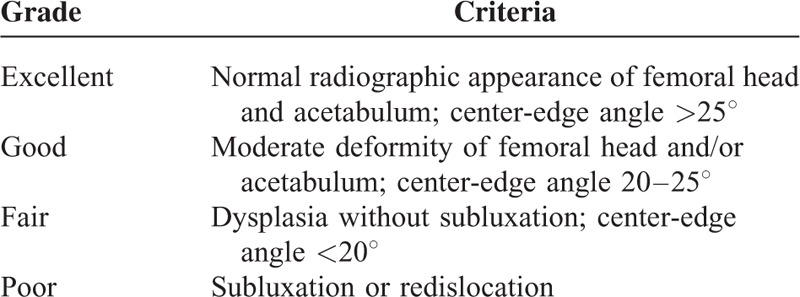
The Modified Severin Criteria for Radiologic Evaluation

### Operative Technique

All index procedures were performed by our senior doctor (Xu Rui-Jiang). The surgical techniques of modified Chiari pelvic osteotomy,^28,29^ Dega osteotomy,^30^ shelf acetabuloplasty,^31^ STR,^32^ and ATPT^20,21^ used in this study were done according to procedures described previously. The indications for acetabuloplasty procedures were an acetabular index (AI) of >25° or a Sharp angle of >45°, and/or a center-edge angle (CE angle) of <20° on the preoperative plain radiographs.^33^ The indications for ATPT were lesions of acetabular or femoral cartilage or both detected by preoperative radiography findings and measured intraoperatively under direct visualization.

Briefly, open adductor tenotomy through a small transverse incision was performed, and an iliofemoral approach through a Bikini curve incision was developed, then tenotomy of the tendinous portion of the straight head of the rectus femoris muscle and the iliopsoas muscle were done in indicated cases. The iliac apophysis was incised in line with the iliac crest. Free any adhesions of the joint capsule from the lateral surface of the ilium and false acetabulum and excise redundant capsule flap. A T-shaped incision was then created to incise the capsule superiorly and anteriorly. If any lesions of acetabular or femoral cartilage or both were visualized, an ATPT was then performed. Initially, debridement of the damaged articular surfaces was made: the cartilage defects excised, sclerotic subchondral bone removed, and multiple drilling through the remaining subchondral bone into the cancellous bone performed. The periosteum was procured from the medial surface of the tibia with the size a little bigger than the cartilage defect area. The periosteum was then sutured to the previously prepared defective area with the cambium layer towards the subchondral bone using resorbable sutures. Then femoral and pelvic osteotomies were performed in indicated cases. After reduction of femoral head, capsulorrhaphy was then performed, if the capsule is loose. Then tendinous portion of the straight head of the rectus femoris muscle was sutured to the capsule just under the anterior inferior iliac spine.

After routine closure, a hip spica cast was applied and worn for 4 weeks. Then a single abduction brace was applied for another 4 weeks for early passive ROM under supervision. After removal of the brace, sequential muscle strengthening exercises and active ROM practice in bed commenced for another 8 weeks. Partial weight bearing commenced at 12 weeks postoperatively. Full-weight walking was not permitted until the grafts were confirmed incorporated radiologically, usually at 4 to 5 months postoperatively.

Data were analyzed using a commercial statistical software package (SPSS statistics 17.0). Differences between the groups were compared using a 2-tailed Student *t* test for normally distributed values. For the comparison of categorical variables, a Chi-squared test was performed; otherwise, the Wilcoxon signed-rank test was used to compare variables. A test result has been regarded as statistically significant for *P* < 0.05.

## RESULTS

The preoperative and latest follow-up clinical statuses are shown in Table 4. At latest follow-up, improvements in pain relief, ROM, and AL were found in both groups. However, more percentage of hips in Group I than Group II had no pain, was finally rated in flexion to at least 80° with 20° of adduction–abduction and internal and external rotation and walked without an assistive device for more than 10 city blocks. Overall, 11 hips (64.7%) in Group I and 4 (26.7%) in Group II had an excellent clinical outcome, and 1 (5.9%) in Group I and 4 (26.7%) in Group II had a poor outcome.

**TABLE 4:**
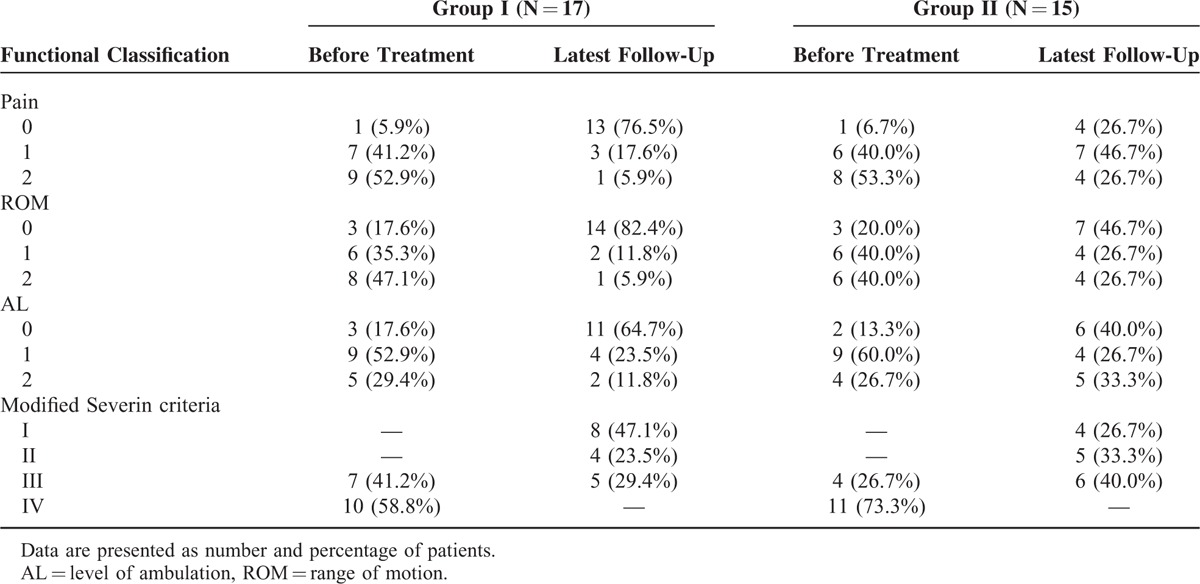
Functional and Radiographic Rating of the Hips

According to the modified Severin criteria, the recent follow-up radiologic evaluation (Table 4) was rated excellent in 8 hips (47.1%) in Group I and 4 hips (26.7%) in Group II, good in 4 (23.5%) in Group I and 5 (33.3%) in Group II; revealing the latest favorable radiologic evaluation was 70.6% in Group I and 60.0% in Group II, respectively. Improvements were found in mean CE and Sharp angles compared between those at latest follow-up and at treatment in both groups (Table 5). The average CE angle improved from −16.7° to 43.3° in Group I and from −17.3° to 45.1° in Group II and average Sharp angle decreased from 54.6° to 35.1° in Group I and from 55.3° to 35.6° in Group II, preoperatively and at latest follow-up, respectively. These parameters showed significant improvement in both groups (*P* < 0.05).

**TABLE 5:**
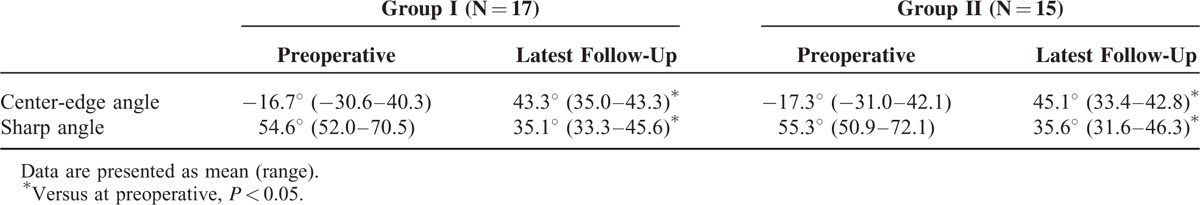
Radiographic Data on the Hips

**FIGURE 1:**
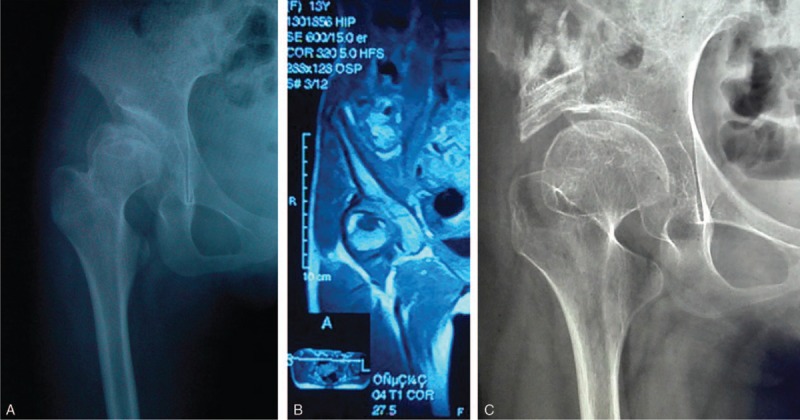
Radiological findings of a 13-year-old girl with a lesion of the right hip, treated with periosteal transplantation combined with surgical reduction of the femoral head and the augmentation of acetabulum. (A) Preoperative anteroposterior hip radiograph showing subluxation in right hip chondral defect in the anteromedial femoral head. (B) Preoperative MRI image was used to confirm location and size of the lesion. (C) Postoperative anteroposterior radiograph depicting well-repaired cartilage defects, good congruency between the femoral head and the shell and well preserved joint space.

**FIGURE 2:**
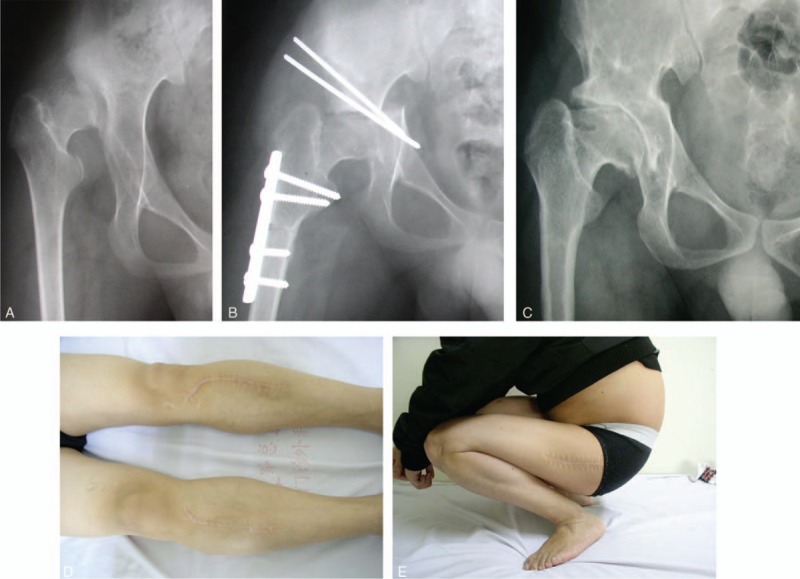
(A) Fifteen-year-old boy. Preoperative hip radiograph showing luxation in right hip. Defects exhibiting in the anterosuperior femoral head. (B) Hip radiograph taken immediately after the periosteal transplantation combined with surgical reduction of the femoral head. (C) Hip radiograph taken 19.3 years after the surgery showing good congruency between the femoral head and the shell and the both joint space well preserved. (D) Photo taken postoperatively showing good wound healing after cutting autologous tibial periosteal. (E) Photo taken 19.3 years after the surgery showing excellent hip range of motion.

Hip radiograph showed incorporated periosteal autograft to the defect area, good congruency between the femoral head and the shell and well-preserved joint space in more hips at latest follow-up in Group I. Whereas more hips exhibited incongruency, deformity of femoral head and acetabulum and narrowed joint space in Group II. Typical radiographic assessments of cases with severe dysplastic hip treated by periosteal autografts transplantation combined with operative procedures are illustrated in Figures 1 and 2.

During the follow-up, few major complications were recorded in Group I. None of the hips developed stiffness or redislocation or ischemic necrosis of the femoral head attributed to index surgery. Only 1 child had to receive subsequent surgery of acetabular augmentation due to residual subluxation following Dega osteotomy. In Group II, 4 poor outcomes with several complications were recorded: residual hip subluxation in 3 hips and avascular necrosis (AVN) in 1 hip. Those hips were subsequently converted to subsequent operation of reefing of joint capsule and/or slotted acetabular augmentation procedure. Persistent pain was encountered in 2 hips, indicating progression of arthritis and possible requirement of total hip replacement. And another hip required a subsequent revision of distal transfer of the greater trochanter.

## DISCUSSION

The efficiency of surgical treatments combined with repair of articular cartilage defects using periosteal autografts for DDH in older children and outcomes after surgery were assessed in this study. The most important novel findings may be that when surgical reduction were chosen to be performed for older DDH children after delayed diagnosis or late presentation of syndromes, repair of articular cartilage defects using periosteal autografts during surgical reduction could be indicated and performed to successfully prevent stiffness, reduce pain and improve ROM and outcomes in the early phase of rehabilitation and long-term follow-up observation.

Surgical intervention to reduce the displaced hip in children aged over 8 years is still controversial. With more complex pathomorphological changes in dysplastic hips, the management of neglected DDH in older children is perplexing and challenging. In general, it is accepted that the ease of treatment and the outcome are inversely related to the age at presentation. Many surgeons recommended that it would be better to forgo surgery for older children with dislocated hips before adulthood^34^ and have hip joint replacement after adulthood. However, if treated negatively, DDH would develop pain limp, hip stiffness, and finally progressive arthritis and disability. Some of the older DDH children would ultimately have to receive total hip arthroplasty even during young adulthood and often more than once during one's lifetime, depending on the severity of the osteoarthritic changes and pain or limitation of motion of the hip they suffer from. This would yield a tremendous social, economic, physical, and emotional impact on older children and their family.

In recent years, researches of the interaction of hip diseases and cartilage lesions are in a rapidly evolving and growing period. It has been demonstrated that cartilage defects are moderately common in dysplastic hips^3–6^ and proved to be highly correlated with hip pain, stiffness, and dysfunction in movement and function.^6–11^ Repair of cartilage defects in the hip has been reported to yield good clinical and radiologic outcomes through osteochondral^7^ or chondrocyte^35^ transplantation. To date, however, a systematic evaluation of the improvement in functionality, physical health, quality of life, and tolerability in association with the cartilage repair after transplantation of periosteal autografts has not yet been performed in dysplastic hip in older children. Given that free periosteal grafts transplantation has the capacity to differentiate into cartilage,^36,37^ this clinical study on the effects of periosteal transplantation in hip rehabilitation in older children with DDH was implemented.

The optimal management of DDH might be early diagnosis, less invasive, and more effective procedures to achieve a stable and concentrically located joint and satisfactory development of the hip in the majority of cases at an early age. However, neglected DDH in older children appears to be constantly at a modestly high prevalence because of late or missed diagnosis, or late presentation of syndromes. Dysplastic hips naturally progress to osteoarthritis in this age patients. When surgical therapy is required because of osteoarthritic progression, the degree of preoperative osteoarthritic involvement plays a decisive role on the consequences of pelvic osteotomy surgery.^38^ Thus, early management of any cartilage abnormalities is important when considering osteoarthritic development and surgical therapy.^6^ Generally, the choice of treatment for large cartilage defects depends on a number of factors, including patient's preference, physician's expertise, and the cost of the procedure.^39^ To adapt the periosteum transplantation for articular cartilage defects, several advantages have been acknowledged. This tissue meets the 3 primary requirements for tissue engineering: a source of cells, a scaffold for delivering and retaining them, and a source of local growth factors. When transplanted into osteochondral articular defects, it has the capacity of producing cartilage to restore the articular cartilage and being replaced by bone in the subchondral region. The succedent research priorities lie in investigating the mechanism of periosteal chondrogenesis at the cellular and molecular levels to optimize its application for cartilage repair and regeneration.^40^

In the hip, due to the restricted joint space, access to the joint is difficult. Exposure of cartilage defects on the femoral head and acetabulum is extremely hard in terms of overall view. This is critical because transplants need further fixation by suture or adhesives. Thus arthrotomies and incision of periosteum are required for approaching the defects, putting the patient at a high risk of postoperative complications and prolonged recovery time. When it comes to patients with dysplastic hips which would be reduced by surgical management, this surgery turns out to be an advantage for exposure and repair of articular cartilage defects during surgical reduction for older DDH children.

Among patients treated with combined acetabuloplasty and ATPT, pain was markedly reduced and hip ROM was improved in all hips including the 2 previously stiff hips during daily life activities. At latest follow-up, higher percentage of patients in Group I had a minimum of 80° of flexion with 20° of adduction–abduction and rotation and all except 2 patients were ambulating without assistive devices. No major concerns but one child developed residual subluxation and required subsequent acetabular augmentation. All children except one continued to function well. All hip showed good congruency between the femoral head and the shell during the visits to outpatient clinic, apart from radiographic sighs of slightly reduced articular space on one hip. The Shenton line kept continuity in most hips. All these results indicate that a very satisfactory functional and radiological outcome could be readily achieved in this older age group of DDH children. In terms of pain relief and improvement of hip function, the periosteal transplantation for articular cartilage defects in older children with DDH seems to be a reasonable adjunctive treatment option for surgical reduction.

Admittedly there are several limitations in our study. Little has been fully elucidated about the exact mechanisms by which periosteal autograft results in clinical improvement in hip rehabilitation. Besides, although there is no consensus statement on the influence of defect size on postoperative outcome, the distribution and size of the defects differ from case to case. A subsequent larger-scale randomized controlled clinical trial and experimental mechanism research at the cellular and molecular levels are needed to address these concerns.

In summary, data from this study would demonstrate that, application of periosteal autograft for the repair of cartilage defects within the hip joint as an adjunctive treatment during surgical reduction procedures in hip rehabilitation might be effective in preventing stiffness, reducing pain and improving ROM and outcomes in the long-term follow-up in older children with DDH. It could be considered as a salvage procedure to hip management in this older and difficult-to-treat group of DDH children.
